# Enhancing Zn(ii) adsorption efficiency of alginic acid through pyridine-2-imine functionalization: kinetic, isotherm, and mechanistic insights

**DOI:** 10.1039/d5ra04669f

**Published:** 2025-10-20

**Authors:** Upma Vaid, Sunil Mittal, J Nagendra Babu, Rimzim Jasrotia, Harminder Singh, Sandeep Kumar

**Affiliations:** a Department of Environmental Sciences and Technology, School of Environment and Earth Sciences, Central University of Punjab Bathinda Punjab 151001 India; b Department of Chemistry, School of Basic Sciences, Central University of Punjab Bathinda Punjab 151001 India; c Chandigarh University Mohali Punjab 140413 India; d School of Chemical Engineering and Physical Sciences, Lovely Professional University Phagwara Punjab 144411 India; e Department of Chemistry, Akal University Talwandi Sabo Bathinda Punjab 151302 India sandeepchem83@gmail.com sunil.cevs@gmail.com +91-98883-88330 +91-98156-20186

## Abstract

This study investigates the functionalization of alginic acid (AA) with pyridine-2-imine (PAA) through a two-step reaction involving coupling and condensation. The objective was to enhance the Zn(ii) adsorption efficiency of alginic acid. The PAA derivative was characterized to assess the extent of functionalization, changes in functional groups, surface morphology, and thermal decomposition behavior. Batch adsorption studies revealed a significant improvement in Zn(ii) adsorption efficiency, with PAA achieving 83.36 mg g^−1^, compared to 44.28 mg g^−1^ for unmodified AA. The adsorption of Zn(ii) on PAA was found to follow the pseudo-second order kinetic model, with equilibrium data fitting both Langmuir and Freundlich isotherms. Mechanistic analysis using FTIR and ^13^C CP-MAS NMR spectroscopy indicated that Zn(ii) binds exclusively to the carboxyl group in AA, while in PAA, it binds to both the amide and imine groups, explaining the enhanced adsorption efficiency. These findings demonstrate that pyridine-2-imine functionalization significantly improves the Zn(ii) adsorption capacity of AA from aqueous solutions. Future research could focus on optimizing the functionalization process and exploring its application in real-world water treatment systems.

## Abstract 2

This study investigates the functionalization of alginic acid (AA) with pyridine-2-imine (PAA) through a two-step reaction involving coupling and condensation. The objective was to enhance the Zn(ii) adsorption efficiency of alginic acid.

## Graphical abstract

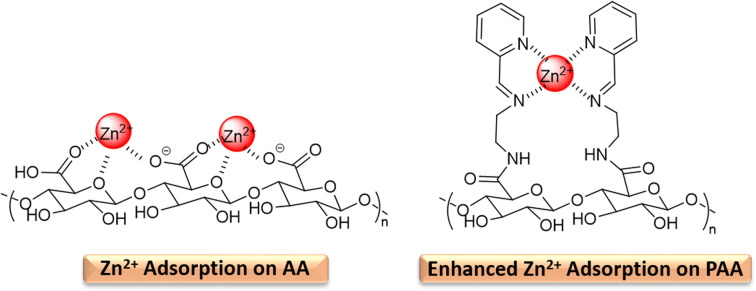

## 1. Introduction

Zinc is a vital trace element that plays a crucial role in numerous biological processes, including enzyme activity, immune function, protein synthesis, and DNA repair. It is essential for growth, development, and cell division, making it indispensable for human health and well-being.^1^ However, excessive concentrations of zinc in the environment, often due to industrial activities like mining, metal production, and agricultural runoff, can lead to toxic contamination of soil and water, negatively affecting ecosystems and human health.^2^ It is among the thirteen metals in contaminant list projected by the United States Environmental Protection Agency.^3^ In aquatic organisms, Zn(ii) not only decline aquatic organisms' memory but also brings dysfunction to their vital organs like pancreas and kidney.^4^ This contaminated water when used for irrigation, Zn(ii) enter into soil where it persists for longer periods of time and can get easily bio-accumulate with food chain.^5^ Zn(ii) when ingested in excess by human beings can causes increased thirst, depression, lethargy, neurologic signs like seizures, ataxia, anemia, *etc.*^6,7^ The maximum acceptable concentration of zinc in drinking water as per Bureau of Indian Standards and World Health Organization (WHO) is 5.0 mg L^−1^.^8^ Hence, sufficient treatment of industrialized discharge is needed before discharging into water bodies.

Currently, the removal of Zn(ii) and other heavy metals from wastewater is typically achieved using various physio-chemical techniques such as precipitation, coagulation, flocculation, chemical oxidation, ion-exchange, membrane filtration, and reverse osmosis.^9–12^ However, these methods have several limitations, including incomplete removal, low efficiency, the need for delicate operating conditions, high energy consumption, the production of large amounts of toxic sludge, and expensive disposal costs.^10,13,14^ To address these issues, ongoing research is focused on developing more effective and economical solutions. This has led to the exploration of various natural, low-cost materials as adsorbents, as adsorption offers an efficient, cost-effective process with simpler operation and maintenance.^15–17^ Studies suggest that for an adsorbent to be ideal for heavy metal removal, it should be readily available, affordable, possess a large surface area, appropriate pore size, mechanical stability, high selectivity, and efficient adsorption capabilities.^18,19^

Efforts to develop optimal adsorbents have increasingly focused on utilizing byproducts derived from bio-macromolecules, as explored in several studies.^20,21^ Among these, alginates have emerged as one of the most abundant biopolymers in nature. They are primarily extracted from the intracellular medium of brown algae species such as *Laminaria hyperborea*, *Laminaria digitata*, *Laminaria japonica*, *Ascophyllum nodosum*, and *Macrocystis pyrifera* through relatively simple methods. This natural abundance, combined with their unique chemical properties, makes alginates an attractive material for various applications, including heavy metal ion removal.^22,23^ Due to their high affinity for metal ions, alginates have been studied extensively for their potential use in water treatment and pollution control, especially for removing toxic metals from industrial effluents. Alginates contain a significant number of free hydroxyl (–OH) and carboxyl (–COOH) groups, which play a crucial role in their ability to complex with a wide range of heavy metals. These functional groups serve as coordination and reactive sites, enabling alginates to effectively bind metal ions.^24^

Moreover, alginates can be easily modified through functionalization, which enhances their performance as adsorbents. Specifically, the introduction of sulfur and nitrogen-containing groups onto the polymer backbone significantly improves their selectivity, reactivity, and binding capacity toward heavy metal ions.^25,26^ Such chemical modifications lead to the development of more effective adsorbent materials with tailored properties, making them more suitable for specific applications, such as the selective removal of certain metal ions from solutions.^27^

This enhancement in the properties of alginates after chemical modification is attributed to changes in the surface characteristics of the polymer. These alterations increase the material's affinity for metal ions and improve its overall adsorption efficiency. Compared to their unmodified counterparts, chemically functionalized alginates exhibit superior performance, which has been well-documented in various studies.^28^ Thus, the functionalization of alginates represents a promising avenue for developing more efficient and targeted adsorbents for environmental remediation applications, particularly in the removal of toxic heavy metals from water systems.^29^

This study presents the synthesis, characterization, and evaluation of a pyridine-2-imine derivative of alginic acid (PAA) for the removal of Zn(ii) ions from aqueous solutions. A two-step synthesis process was employed to modify the structure of alginic acid by incorporating the pyridine-2-imine functional group. The structural properties of the synthesized PAA were confirmed through various characterization techniques, including FTIR and NMR spectroscopy. Comparative adsorption experiments revealed that PAA exhibited significantly higher adsorption capacity for Zn(ii) ions compared to unmodified alginic acid (AA), highlighting the enhancement imparted by the pyridine-2-imine modification. The adsorption mechanism was further explored, showing that both electrostatic interactions and complexation through functional groups play key roles in the improved binding of Zn(ii). This research demonstrates the potential of functionalized alginic acid derivatives, such as PAA, as efficient and selective adsorbents for the removal of heavy metal ions, offering promising applications in environmental remediation.

## 2. Materials and methods

### 2.1. Reagents and materials

The chemical reagents, including zinc nitrate hexahydrate (98%), sulfuric acid (98%), sodium hydroxide (98%), sodium alginate (91%), ethylene diamine (98%), *N*,*N*′-dicyclohexyl carbodiimide (98%), dimethylformamide (99%), *N*-hydroxysuccinimide (98%), ethanol (99.9%), chloroform (99%), methanol (99%) were of analytical grade purchased from Loba Chemie Pvt Ltd India. The pyridine-2-aldehyde (99%) was purchased from Sigma-Aldrich.

### 2.2. Synthesis of alginic acid derivatives

Alginic acid (AA) was synthesized by acidifying sodium alginate.^30^ The carboxyl content (m eq-COOH/100 g sample) of AA was determined using the acid–base titrimetric method.^31^ This alginic acid was then derivatized in two stages.

#### 2.2.1. Synthesis of amino-amide conjugated AA

In the first step, the AA (2.2 g) was placed in a round-bottom flask (RBF) and reacted with ethylenediamine (6.0 g), *N*,*N*′-dicyclohexyl carbodiimide (DCC, 2.8 g) as a coupling agent, and dried dimethylformamide (DMF, 10 mL) as the solvent. To prevent by-product formation, *N*-hydroxysuccinimide (NHS, 1.6 g) was added. The reaction was allowed to stir at room temperature for 24 h. After completion, the chloroform was added to terminate the reaction, and the product was filtered and washed extensively with chloroform. The resulting product was then washed twice with ethanol and air-dried overnight in hot air oven.^28^

#### 2.2.2. Synthesis of amino-amide tapered AA *via* Schiff's base formation

In the second step, the Schiff base, pyridine-2-imine derivative of alginic acid (PAA), was synthesized by reacting the first-step product (AA, 2.24 g) with pyridine-2-aldehyde (0.96 mL). This reaction was carried out under continuous stirring for 24 h in methanol as the solvent. The PAA was then thoroughly washed with methanol and air-dried overnight.

#### 2.2.3. Impact of reaction conditions (temperature and time) on PAA formation and quality

Both steps of the AA → PAA functionalization are sensitive to thermal history and reaction time. In the DCC/NHS-mediated coupling (AA + ethylenediamine, DMF), mild temperatures (20–25 °C) and 18–24 h typically favor amide formation while minimizing side reactions (*e.g.*, *O*-acylurea/*N*-acylurea formation) and alginate backbone scission. Prolonged times (>24–36 h) or higher temperatures can promote undesired crosslinking/gelation and reduce solubility/processable yield, ultimately lowering the number of accessible –NH_2_ sites for the subsequent condensation. Conversely, insufficient time (<12 h) can limit conversion and lead to lower nitrogen incorporation. In the Schiff-base condensation (first-step product + pyridine-2-aldehyde, MeOH), room temperature with ∼24 h stirring provides an effective balance between imine formation and avoidance of hydrolysis in protic media. Slight increases in temperature can accelerate imine formation but also increase the risk of hydrolysis/rearrangement if water accumulates; therefore, dry solvent and tight control of time are advisable.

### 2.3. Characterization of AA and PAA

The elemental composition (N, C, H, and O) of both AA and PAA was determined using elemental analysis (CHNO) conducted on a Flash 2000 Thermo Elemental Analyzer. To examine the elemental makeup of AA and its derivative PAA, Field Emission Scanning Electron Microscopy (FE-SEM) with a ZEISS Merlin Compact and Energy Dispersive Spectroscopy (EDS) with an Oxford X MAXn detector were employed. The FTIR spectra of AA, PAA, and their Zn(ii)-adsorbed forms were recorded using a BRUKER TENSOR 27 spectrometer, which helped identify the changes in functional groups during derivatization and the key groups involved in metal ion binding. For structural analysis, Cross-Polarization Magic Angle Spinning Carbon-13 Solid-State Nuclear Magnetic Resonance (^13^C CP MAS SS-NMR) was performed on a Bruker Avance II (500 MHz) system. Thermo-gravimetric analysis (TGA) and Differential Thermal Analysis (DTA) of AA, PAA, and their Zn(ii)-adsorbed counterparts were conducted using a Shimadzu DTG-60H thermo-gravimetric analyzer. The samples were heated from 30 to 500 °C at a rate of 10 °C min^−1^ in an alumina pan under a nitrogen atmosphere (flow rate of 40 mL min^−1^).

### 2.4. Adsorption experiments

Batch adsorption experiments were conducted to investigate the effects of various parameters, including pH, contact time, adsorbent dose, and initial ion concentration, on the adsorption efficiency of Zn(ii) by AA and PAA. Initially, the pH was adjusted by shaking 100 mL of a 100 mg L^−1^ Zn(ii) solution with 0.5 g L^−1^ of AA or PAA at different pH values (3–5) for 2 h at 25 ± 1 °C and 200 rpm. After reaching equilibrium (22 h), the solution was filtered through Whatman filter paper, and the metal ion concentration in the aqueous phase was measured using a stripping voltmeter (Model: Metrohm, 797 VA COMPUTRACE).

The effect of sorbent dosage on Zn(ii) adsorption was studied by shaking different amounts of sorbent (0.25, 0.5, 1.0, and 2.0 g L^−1^) with 100 mL of 100 mg L^−1^ Zn(ii) solution at pH 5. The impact of contact time (2.5, 5, 10, 15, 30, 60, and 120 min) on the adsorption kinetics of Zn(ii) by AA and PAA was analyzed by shaking 0.25 g L^−1^ of the adsorbent with 100 mL of the 100 mg L^−1^ Zn(ii) solution at pH 5 for varying durations at 200 rpm. The effect of initial metal ion concentration (20, 40, 60, 80, 100, 120, and 140 mg L^−1^) was investigated by shaking 0.25 g L^−1^ of adsorbent with 100 mL of metal ion solutions at pH 5 for 2 h.

The percent removal of Zn(ii) (*R*%) and the sorption capacity (*q*_e_) of AA and PAA were calculated using the data from the above experiments, following the equations eqn (1) and (2) respectively.1
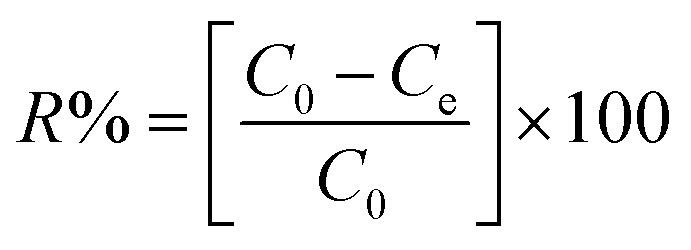
2
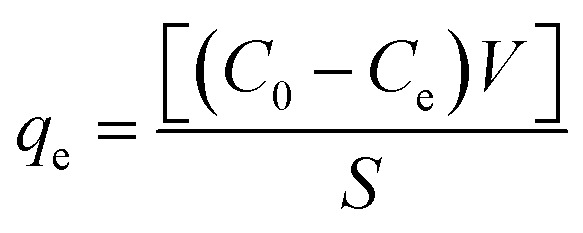
where, *C*_0_ and *C*_e_ is the initial and final concentration of Zn(ii) ions (mg L^−1^) in aqueous solution, respectively. *S* is mass of adsorbent (in g), and *V* states the volume of aqueous solution treated (in L).

The Langmuir and Freundlich adsorption isotherm models were applied to the experimental data obtained to investigate the Zn(ii) removal efficiencies of AA and PAA, and to elucidate the underlying mechanism of removal. The Langmuir isotherm assumes that adsorption occurs on a uniform surface with a fixed number of reversible sites. The equation for the Langmuir isotherm is expressed in eqn (3) as follows:3
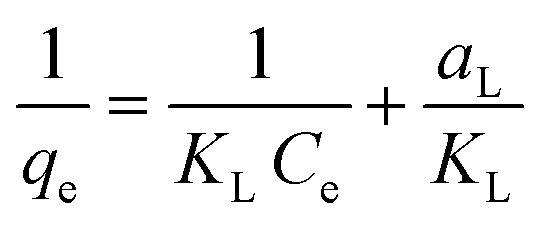
where, *K*_L_ represents the Zn(ii) adsorptivity (L g^−1^), and *a*_L_ (L mg^−1^) is related to the energy of adsorption.

The Freundlich adsorption isotherm describes adsorption on a heterogeneous surface with a non-uniform distribution of adsorption sites, and is expressed as eqn (4) as follows:

4 ln *q*_e_ = *b*_F_ ln *C*_e_ + ln *K*_F_

where, *K*_F_ represents the adsorbent capacity (L g^−1^), and *b*_F_ is the unitless heterogeneity factor ranging from 0 to 1.^32^

## 3. Result and discussion

### 3.1. Characterization of AA and PAA

**Scheme 1:**
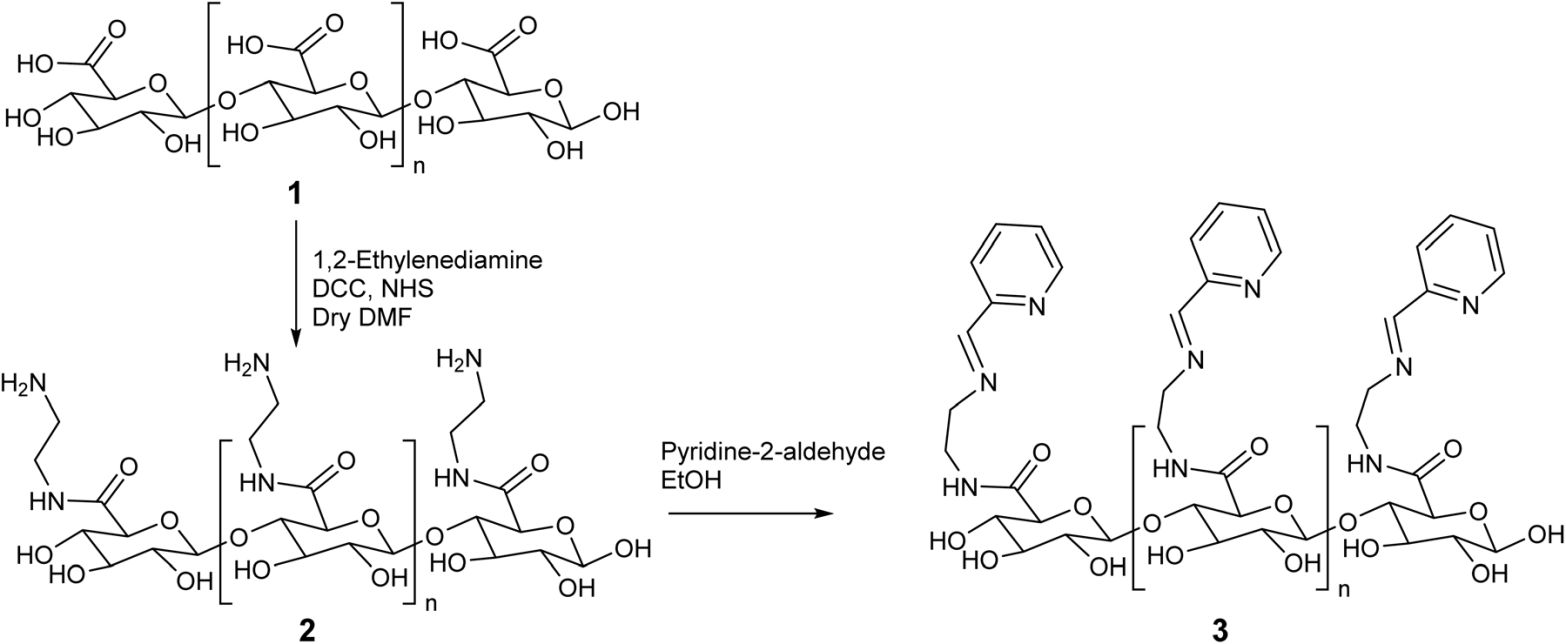
Schematic representation for synthesis of PAA.

The acid–base titrimetric analysis of synthesized AA indicated that approximately 85 ± 3% of the Na + ions in sodium alginate were replaced by protons during the synthesis of AA, which contained roughly 495.5 meq COOH/100 g of the sample.^11^ The preparation of the compound PAA involved a two-step process: first, the coupling of AA with 1,2-ethylenediamine,^28^ followed by a condensation reaction between the product from step one and pyridine-2-aldehyde. The synthesis route of PAA is illustrated in Scheme 1. Both AA and its derivative (PAA) were analyzed using various techniques, including elemental analysis (for N, C, H), FTIR, and ^13^C CP-MAS NMR. The elemental analysis results for AA (Table 1) were consistent with those reported.^33^ Additionally, the presence of 42% nitrogen in PAA serves as a key indicator of the successful coupling and condensation of AA with pyridine-2-aldehyde. The empirical formulas for AA and PAA, derived from CHNSO analysis, were C_6_H_8_O_6_.H_2_O for AA and C_12.2_H_16.1_N_2.7_O_5.4_H_2_O for PAA. The empirical formula for PAA also suggested that 70% of the Schiff's base condensation occurred, leaving 30% of the amino groups unreacted. This incomplete condensation may be attributed to the heterogeneous nature of the condensation reaction on the polymer surface, where the core amine groups remained unreacted.

**Table 1:** Percent CHNO composition of AA and PAA

Elements	Percentage composition			
	AA		PAA	
	Theoretical	Experimental	Theoretical	Experimental
C	40.909	35.958	54.723	41.185
H	4.545	5.017	5.537	5.974
N	—	—	13.68	10.513
O	54.545	59.025	26.058	42.026
Formula	C_6_H_8_O_6_	C_6_H_8_O_6_·H_2_O	C_14_H_17_N_3_O_5_	C_12.2_H_16.1_N_2.7_O_5_·3H_2_O

**Fig. 1:**
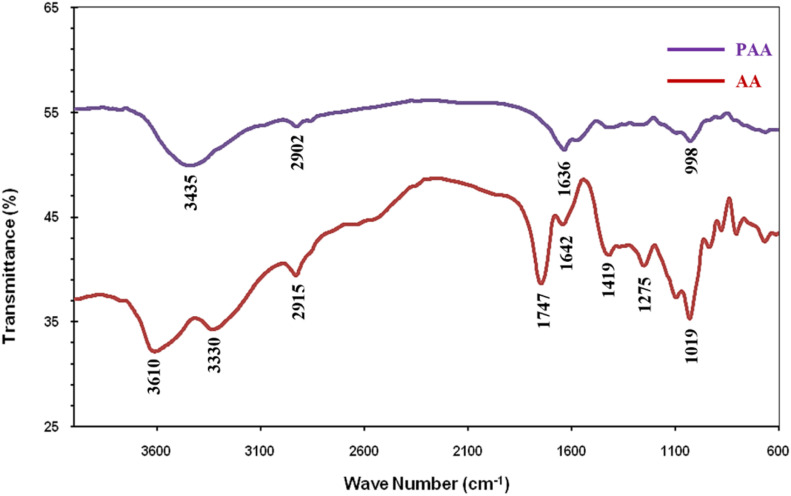
FTIR spectrum of AA and PAA.

The FTIR spectra of AA exhibited absorption peaks at 3610, 3330, 2915, 1747, 1642, 1419, 1275, 1019 cm^−1^. The broad bands observed at 3610 and 3330 cm^−1^ are ascribed to O–H stretching due to intermolecular and intramolecular type hydrogen bonding, respectively. The characteristic band for carboxylic acid at 1747 cm^−1^, carboxylate ion at 1642 cm^−1^ and 1419 cm^−1^ were also present. The band at 2915 cm^−1^ was attributed to alkyl C–H stretching. The absorption bands at 1642 cm^−1^ and 1419 cm^−1^ were assigned to the asymmetrical and symmetrical C–O stretching vibrations of COO^−^ in alginic acid.^34^ Peaks observed at 1275 and 1019 cm^−1^ are observed due to bending vibrations of C–O–C group of pyranose ring.^35^ The Schiff base condensation of AA with pyridine-2-aldehyde results in significant shift in the absorption bands. The peaks observed at 3435 cm^−1^ and 1636 cm^−1^ in case of PAA are characteristic of Schiff base condensation of AA with pyridine-2-aldehyde. Peak at 1636 cm^−1^ observed in case of PAA was broad corresponding to the presence of imine and amide functional moieties of PAA^36^ (Fig. 1).

**Fig. 2:**
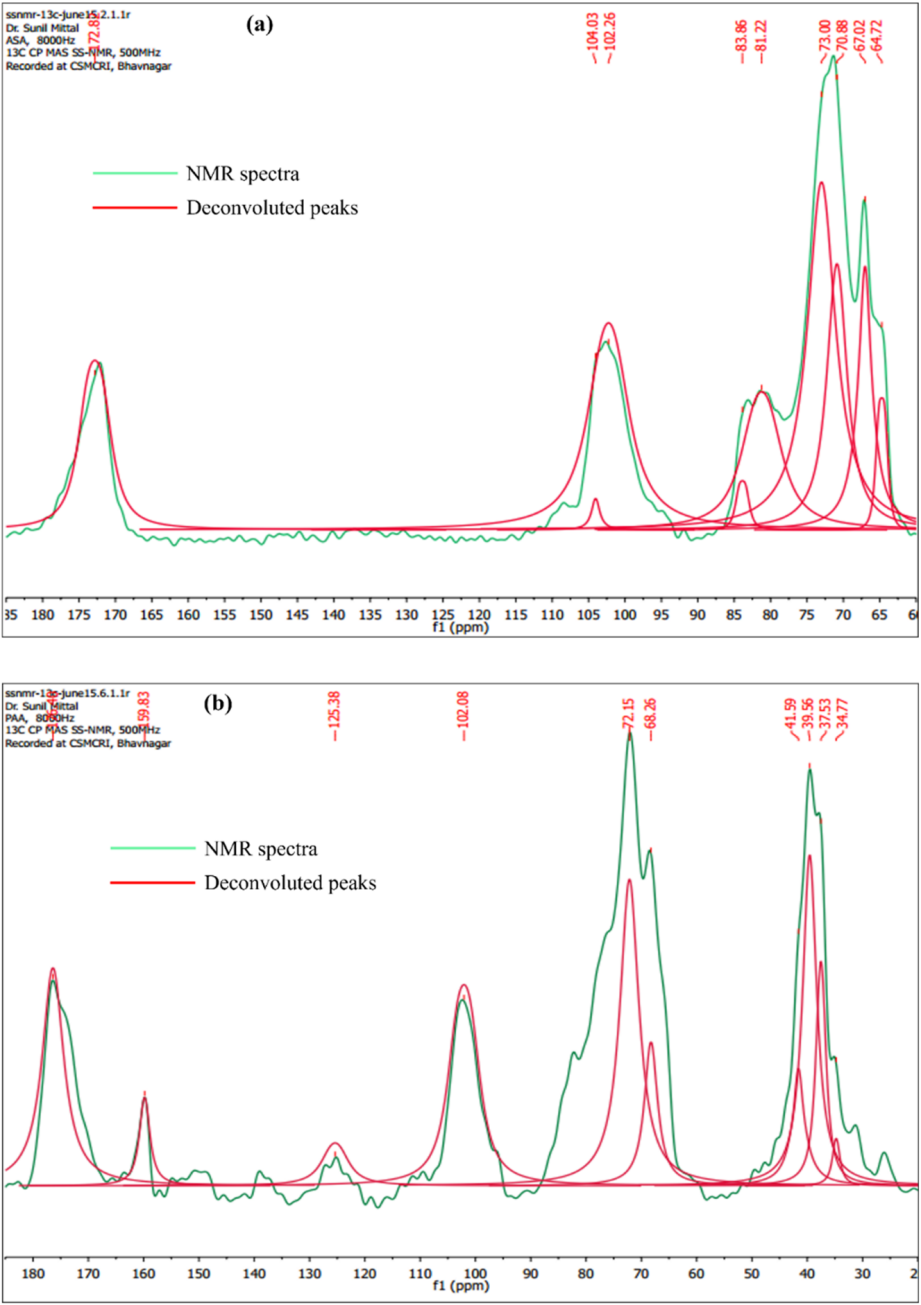
Global Spectral Deconvolution of CP-MAS ^13^C NMR spectrum of (a) AA; and (b) PAA.

Fig. 2a and b presents the Global Spectral Deconvolution of CP-MAS ^13^C NMR spectra of AA and PAA from *δ* 20–185 ppm. In the CP-MAS ^13^C NMR spectra of AA, the peaks appeared in the range around 65–80 ppm are alkyl carbons from the polymer backbone, and peak appeared at 172 ppm indicate the presence of carboxyl carbons (Fig. 2a).^37^ In the CP-MAS ^13^C NMR spectra of PAA, a peak characteristic of aromatic carbons of pyridine was observed at *δ* 125.38 ppm.^38^ Apart from this, new peaks were observed at *δ* 176.48 ppm merged with the existing amide peak, which is characteristic of imine condensation of PAA (Fig. 2b).^39^

**Fig. 3:**
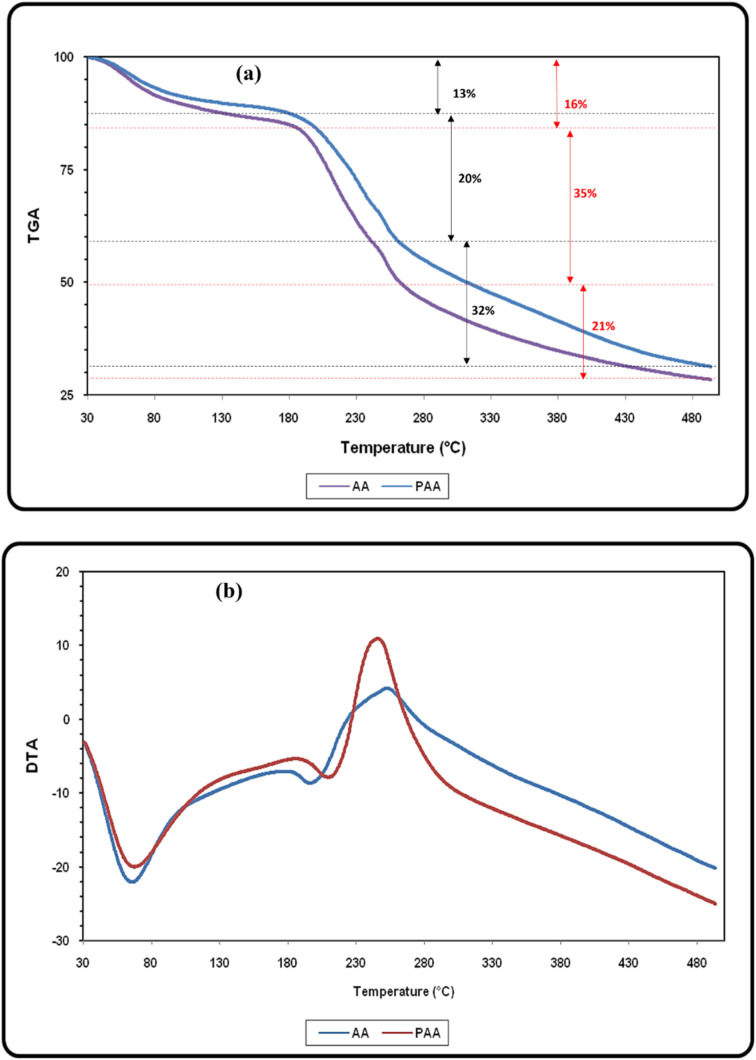
(a) Thermo-gravimetric analysis (TGA); and (b) differential thermal analysis (DTA) of AA and PAA.

This initial weight loss in AA and PAA in temperature range 30 to 180 °C is due to the removal of adsorbed water and volatile organic compounds from the samples. A slight degradation of the alginic acid framework also begin in this region. In the region 180 to 480 °C, the major thermal degradation of alginic acid occurs (Fig. 3a). The polymer backbone of AA and PAA including polysaccharide structure, carboxyl groups (–COOH) and other functional groups begins to break down and undergoes pyrolysis, resulting in the release of gases such as carbon dioxide (CO_2_), carbon monoxide (CO), and other organic volatiles.^34^ This weight loss in the temperature range 200–350 °C with exothermic peak maxima at 245 °C and 255 °C in the DTA graph of AA and PAA, respectively was attributed to the loss of carbonylic, and pyrone structures (Fig. 3b).^40^ The slow weight loss after 330 °C may be ascribed to formation of more stable residue *i.e.* biochar, which decomposes very slowly.

**Fig. 4:**
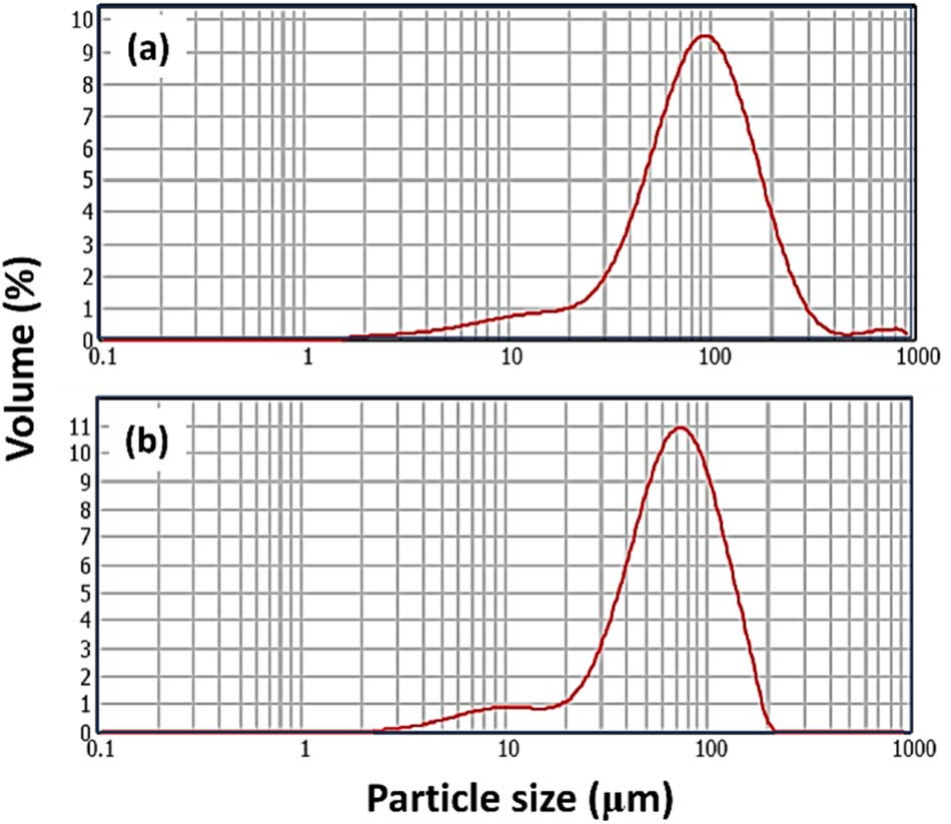
Particle size distribution of (a) AA, and (b) PAA.

The particle size distributions of AA, and PAA are presented in Fig. 4. Analysis of particle size indicated mean values of 41.243 μm for AA, and 66.840 μm for PAA. In addition, their specific surface areas were calculated as 0.244 m^2^ g^−1^, and 0.140 m^2^ g^−1^, respectively. The corresponding uniformity coefficients were determined to be 0.620 for AA, and 0.461 for PAA.

### 3.2. Batch adsorption study

#### 3.2.1. Effect of pH and adsorbent dosage

**Fig. 5:**
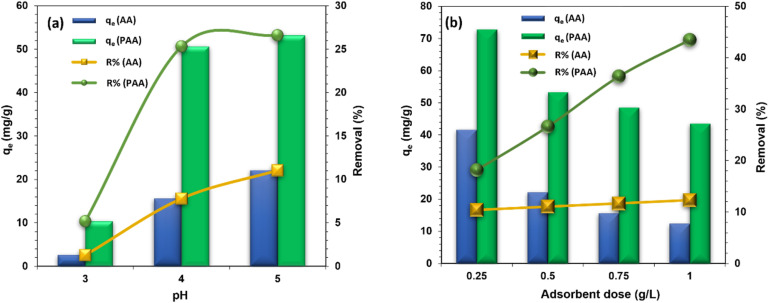
Effect of (a) pH; and (b) adsorbent dose on the removal of Zn(ii) by AA and PAA. {[Zn^2+^] = 100 mg L; [adsorbent] = 0.5 g L; temp. = 25 ± 1 °C, time = 2 h, shaking speed = 200 rpm, equilibrium time = 22 h}.

As the pH rises from 3 to 5, the uptake of Zn(ii) ions by AA and PAA increases significantly, with values rising from 2.58 mg g^−1^ to 22.08 mg g^−1^ for AA and from 10.38 mg g^−1^ to 53.24 mg g^−1^ for PAA (Fig. 5a). Similarly, the percentage of Zn(ii) removal efficiency for AA and PAA increases from 1.29% to 11.04% and from 5.19% to 26.62%, respectively. This increase in adsorption efficiency with rising pH can be attributed to the protonation of the adsorbents. At lower pH levels, the concentration of hydronium or proton ions is high, which competes with the metal ions for binding to the active sites, such as the carboxylate sites on AA.^28,41^ Additionally, the carboxyl groups on AA remain protonated at low pH, preventing the metal ions from binding to the active sites, resulting in the metal ions staying in solution. However, as the pH increases from 3 to 5, the deprotonation of carboxyl groups introduces a negative charge on the surface, which leads to electrostatic attraction between the metal ions and the adsorbent surface.^30,31^ This allows the carboxylate ions to bind to the Zn(ii) ions, forming chelate complexes on the surface.^42^

A similar trend was observed with PAA, where Zn(ii) uptake and removal efficiency increased with pH. At low pH, proton ions compete with Zn(ii) ions for binding to the pyridyl nitrogen of PAA. The high concentration of H^+^ ions at lower pH causes the NH_2_ groups on PAA to become protonated, forming pyridinium ions that give the surface a positive charge. This positive charge at low pH results in repulsion between PAA and Zn(ii) ions, leading to lower adsorption. In contrast, at higher pH, deprotonation of the NH_2_ groups enhances the uptake of Zn(ii) ions.^43^ Since the maximum uptake of metal ions and removal efficiency were observed at pH 5.0 for both adsorbents, pH 5.0 was chosen as the optimal pH for the adsorption process.

The experimental results, shown in Fig. 5b, demonstrate that increasing the adsorbent dose from 0.25 g L^−1^ to 1.0 g L^−1^ leads to an increase in Zn(ii) removal efficiency. For AA, the removal efficiency rises from 10.39% to 12.33%, while for PAA, it increases from 18.18% to 43.51%. This improvement in removal efficiency is attributed to the greater surface area and the increased number of active adsorption sites on the adsorbent.^44^ However, the amount of Zn(ii) adsorbed per gram of adsorbent (*q*_e_) decreases for both AA and PAA as the adsorbent dose increases. Specifically, qe decreases from 41.56 mg g^−1^ to 12.33 mg g^−1^ for AA and from 72.71 mg g^−1^ to 43.51 mg g^−1^ for PAA as the dose is increased from 0.25 g L^−1^ to 1.0 g L^−1^ (Fig. 5b). This decrease in *q*_e_ with higher adsorbent doses may occur because a larger amount of adsorbent results in a lower concentration of metal ions per unit mass of adsorbent, leaving many active sites unsaturated during the adsorption process.^45^

#### 3.2.2. Effect of initial Zn(ii) conc. and adsorption equilibrium studies

**Fig. 6:**
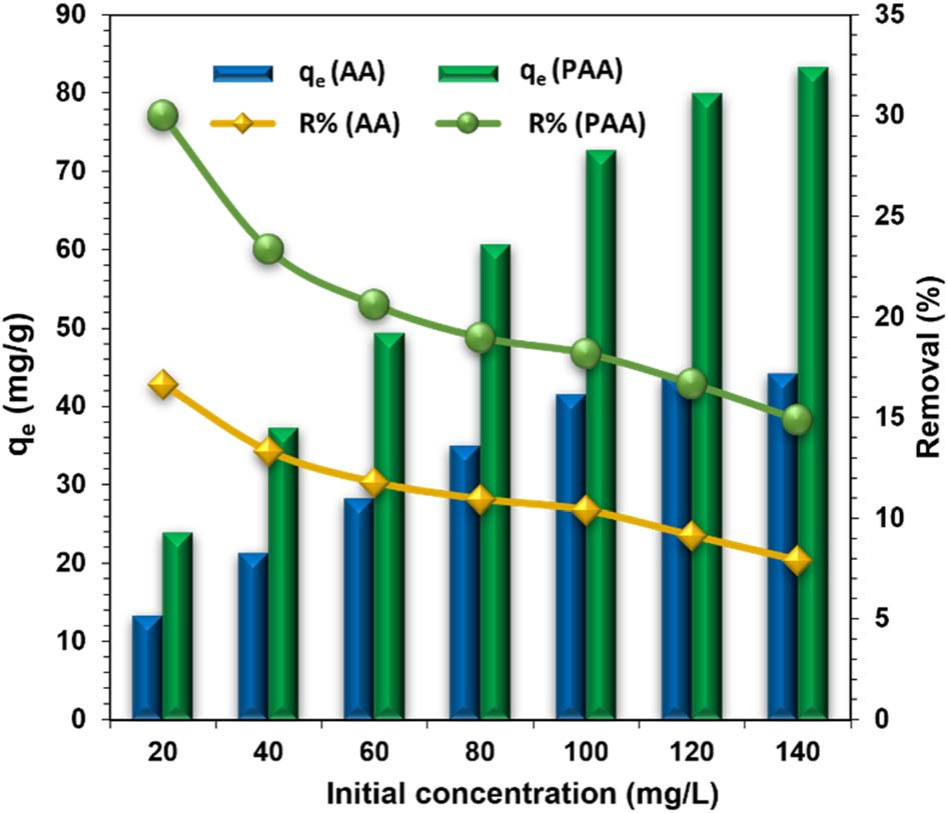
Effect of change in initial Zn(ii) concentrations the adsorptive removal efficiencies of AA and PAA. {[Adsorbent] = 0.25 g L^−1^, pH = temp. = 25 ± 1 °C, time = 2 h, shaking speed = 200 rpm, equilibrium time = 22 h).

The effect of initial Zn(ii) ion concentration on its uptake by AA and PAA was investigated by varying the concentration from 20 mg L^−1^ to 140 mg L^−1^. The results, shown in Fig. 6, reveal that as the initial Zn(ii) ion concentration increased, the metal ion uptake also increases from 13.32 mg g^−1^ to 44.28 mg g^−1^ for AA, and from 24.00 mg g^−1^ to 83.36 mg g^−1^ for PAA. However, the percentage removal of Zn(ii) decreased as the concentration increased, dropping from 16.65% to 7.91% for AA and from 30% to 14.89% for PAA. The gradual decrease in the percentage removal of Zn(ii) for both AA and PAA with increasing concentrations can be attributed to the higher number of Zn(ii) ions competing for a limited number of available surface adsorption sites on the adsorbents.

**Fig. 7:**
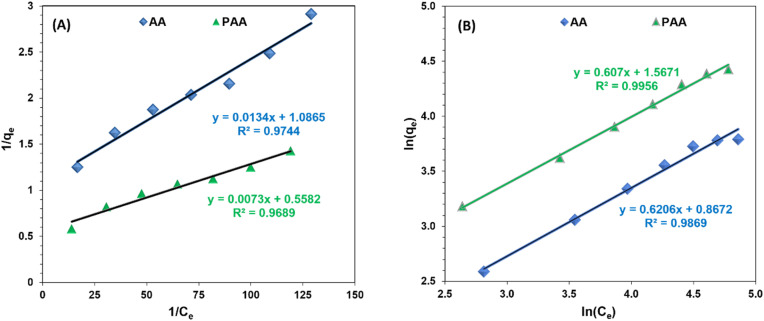
Fitting of Zn(ii) adsorption on AA and PAA using (a) Langmuir adsorption isotherm; and (b) Freundlich adsorption isotherm models.

The equilibrium adsorption data demonstrated a good fit for both the Langmuir and Freundlich adsorption isotherm models (Fig. 7a and b), indicating that the adsorption behavior of PAA is consistent with monolayer adsorption of Zn(ii). The Langmuir model provided the following values: *q*_m_ = 142.86 mg g^−1^, *b* (adsorption energy) = 0.0126 L mg^−1^, and *R*_L_ (dimensionless factor) = 0.400–0.850. The *R*_L_ value, which lies between 0 and 1, suggests that Zn(ii) adsorption onto PAA is favorable.^46^ In addition, the Freundlich model yielded *K*_f_ and ‘1/*n*’ values of 4.79 and 1.65, respectively. Since the value of ‘*n*’ is greater than 2, it indicates strong adsorption of Zn(ii) onto the PAA adsorbent.^47^

While the Langmuir model assumes a homogeneous surface with a finite number of identical sites, this idealization may not fully capture the heterogeneous nature of PAA, where imine, amide, and residual carboxylate groups contribute differently to Zn(ii) binding. Conversely, the Freundlich model better reflects surface heterogeneity but lacks a true saturation capacity, making it less predictive at high concentrations. In this study, the superior fit of the Langmuir model suggests that monolayer adsorption dominates under the tested conditions, but the coexistence of multiple functional groups indicates that some degree of surface heterogeneity must also be considered.

#### 3.2.3. Effect of contact time and adsorption kinetic studies

**Fig. 8:**
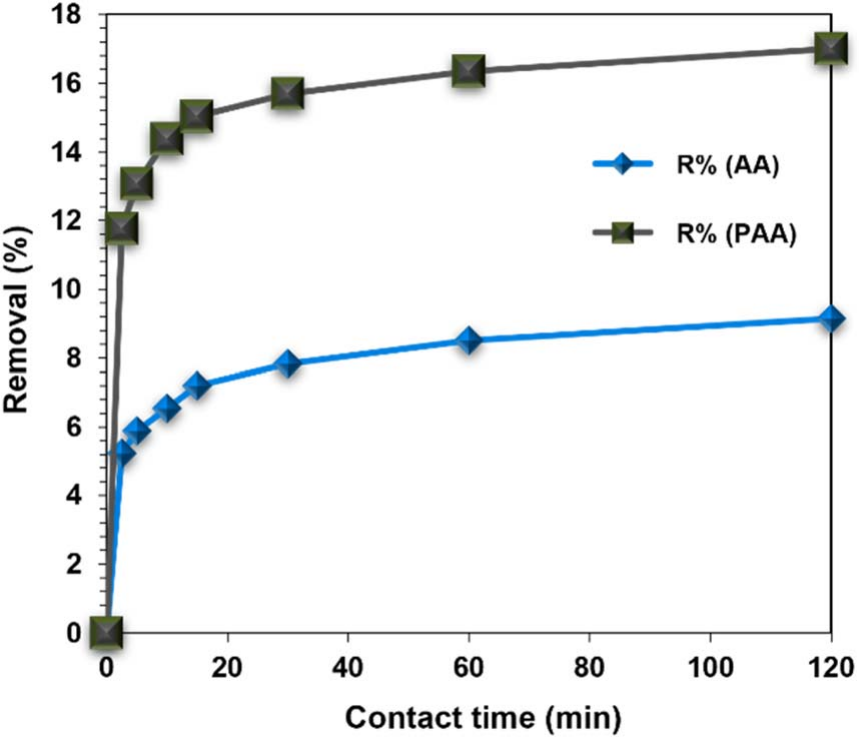
Effect of contact time on % removal of Zn(ii) by AA and PAA ([Zn(ii)] = 100 mg L^−1^, pH = 5, [Adsorbent] = 0.25 g L^−1^, temp. = 25 ± 1 °C, shaking speed = 200 rpm).

Contact time effect on Zn(ii) adsorption has been observed by varying contact time from 0 to 120 min and it was found that the adsorption process is very fast in first 2.5 min and then become slower subsequently till the equilibrium was attained. The removal of Zn(ii) by AA and PAA increased from 5.23 to 9.15% and 11.76 to 17.0%, respectively, upon extending the time of contact from 2.5–120 min (Fig. 8). This may occur due to a fact that at initial stages, there is an accessibility of ample vacant sites near the surface. Therefore, less interruption for upcoming metal ions is there during initial stages.^48^

**Fig. 9:**
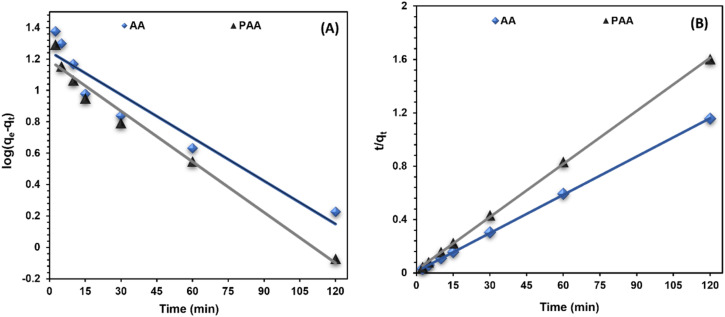
Lagergren pseudo-first order; and (b) pseudo-second order kinetic plot for Zn(ii) adsorption on AA and PAA.

Linear plots for Lagergren pseudo first-order as well as pseudo-second-order kinetic models has been obtained by fitting these models to the obtained data from the experimental study being carried out to know the contact time effect variation (Fig. 9a and b). Both *q*_e_ and rate constants were found by the linear plot between them and the values are presented in Table 2. The value of *q*_e_ drawn from pseudo-second order kinetic plot is in agreement with experimental *q*_e_ for both adsorbents as well as the correlation coefficient value is also high for the same model.

**Table 2:** Comparison of adsorption isotherms and kinetic plot for Zn(ii) adsorption on AA and PAA

Models	Parameter	AA	PAA
**Adsorption isotherm models**			
Langmuir	*q*_max_ (mg g^−1^)	74.63	136.99
	*b* (L mg^−1^)	0.0123	0.0131
	*R*_L_	0.3867–0.8298	0.3905–0.8450
	*R* ^2^	0.974	0.968
Freundlich	*K*_f_ (mg g^−1^)	2.3802	4.7927
	1/*n*	0.6206	0.6070
	*R* ^2^	0.986	0.995

**Adsorption kinetic models**			
PFO	*q*_max_ (mg g^−1^)	41.56	72.72
	*q*_1_ (mg g^−1^)	16.90	17.06
	*k*_1_ (min^−1^)	0.0092	0.0115
	*R* ^2^	0.0092	0.987
PSO	*q*_2_ (mg g^−1^)	37.83	69.12
	*k*_2_ (min^−1^)	0.0065	0.0072
	*R* ^2^	0.998	0.999

The good agreement with the pseudo-second-order model suggests that chemisorption is the dominant mechanism, where electron sharing or exchange occurs between Zn(ii) ions and active sites (imine, amide, and carboxylate groups). This indicates that surface complexation, rather than simple physical adsorption, controls the overall rate. However, at the initial stages of uptake, film diffusion and intra-particle diffusion may also influence transport, particularly as ions move through the porous network of the biopolymer. Thus, while chemical binding at functional groups is the principal rate-limiting step, diffusional resistance could contribute under higher concentrations or at shorter contact times.

### 3.3 Evaluation of the mechanism involved in process

**Fig. 10:**
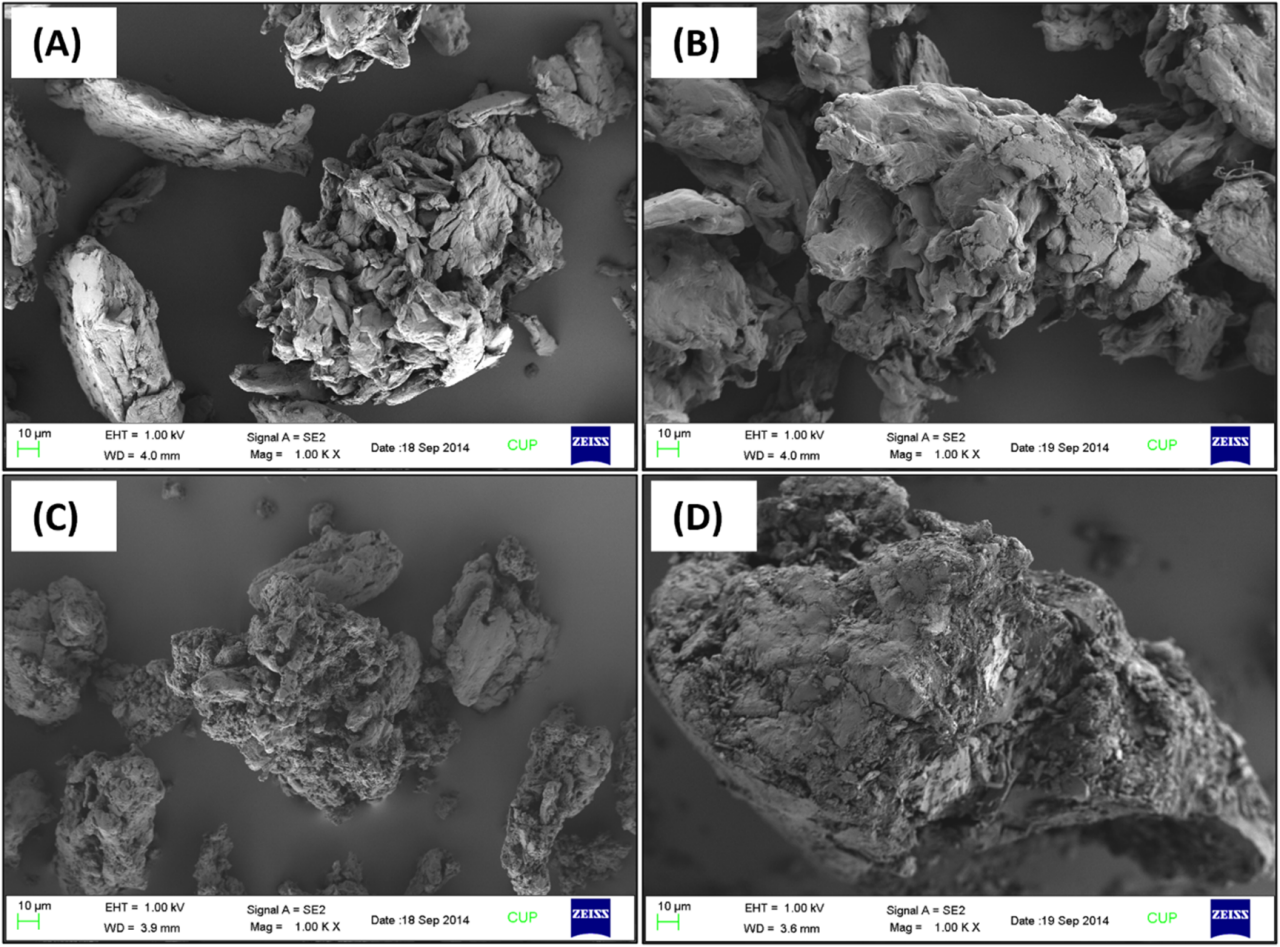
FESEM of AA (a) before, and (b) after; and of PAA (c) before and (d) after, Zn(ii) adsorption studies.

The analysis of SEM and EDX of AA, PAA and their adsorbed Zn(ii) counterparts revealed the variations in surface morphology as well as in elemental composition (%) of adsorbents after Zn(ii) adsorption. Field emission scanning electron micrograph of the AA (Fig. 10a) showed the fibrous and porous structure of AA while, micrograph of adsorbed Zn(ii) AA presented hard arrangement which have less porosity (Fig. 10b). Similarly, PAA (Fig. 10c) images reveal its small and porous structure while, FESEM micrograph of Zn(ii) adsorbed PAA showed large sized and hard structure with low porosity. The changes observed in the morphology of AA and PAA could be attributed to filling of the pore spaces with Zn(ii) ensuing in compact structure (Fig. 10b and d). Table 3 represents weight percent of various element present in AA, PAA and their adsorbed Zn(ii) counterparts derived from EDX analysis. Zn(ii) adsorbed AA and PAA showed the 5.08% and 8.57% Zn, respectively, which indicated that approximately 50.8, 85.7 mg of Zn(ii) was adsorbed per 1 g of AA and PAA, respectively.

**Table 3:** Elemental composition of AA, PAA and their adsorbed Zn(ii) counterparts as given in EDX analysis

Element	Elemental composition (%)			
	AA	Zn(ii)_AA	PAA	Zn(ii)_PAA
C	44.52	40.57	48.23	51.12
O	54.65	53.76	30.43	30.29
Na	0.82	0.59	0.10	0.42
N	—	—	21.24	9.60
Zn	—	5.08	—	8.57

**Fig. 11:**
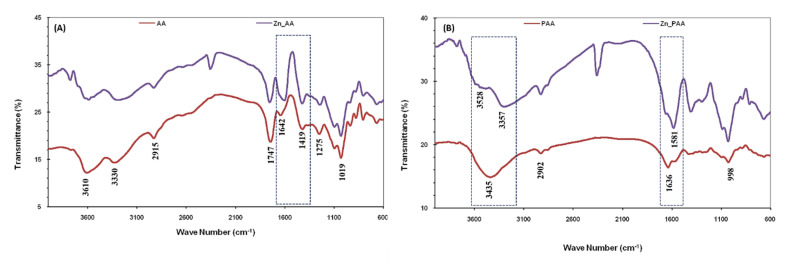
FTIR spectrum of: (a) AA, before and after Zn(ii) adsorption; (b) PAA, before and after Zn(ii) adsorption.

Comparative analysis of FTIR spectrum of AA and Zn(ii) adsorbed AA showed that there are substantial changes observed in range of 1600–650 cm^−1^ which point to straight contribution of the carboxyl group with in the metal-alginate complex (Fig. 11a). On the adsorption of Zn(ii), the peaks shown due to the presence of COO^−^ group of AA present at 1419 and 1642 cm^−1^ in the FTIR spectra shifted to 1411 and 1589 cm^−1^, respectively which can be due to the metal and oxygen bonding of carboxylate group which resulted into change in distance of C–O of the carboxylate group leading to a shift of symmetric peak.^49^ Similarly, comparison of FTIR spectra of PAA with spectra of Zn(ii) adsorbed PAA revealed a significant interaction between PAA and Zn(ii) ions (Fig. 11b). The absorption peak observed at 3435 cm^−1^ in case of PAA was observed to be split into two broad peaks with maxima at 3528 and 3357 cm^−1^, after metal ion adsorption. This characteristic change indicated the involvement of amide in the metal binding and adsorption phenomenon. The absorption band at 1636 cm^−1^ was observed to shift to 1581 cm^−1^, which indicated a significant interaction between Zn(ii) and amidic NH.

**Fig. 12:**
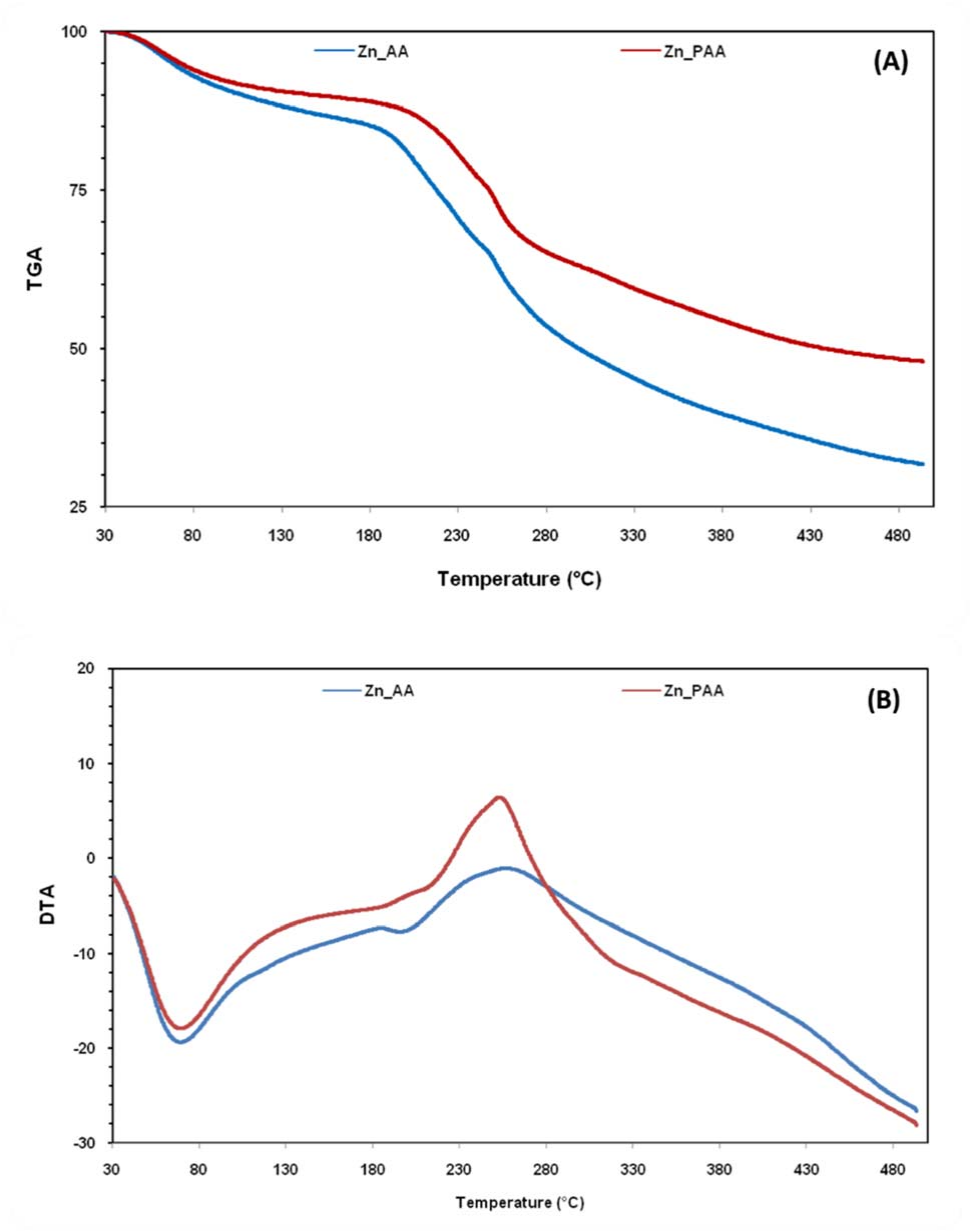
(a) Thermo-gravimetric analysis; and differential thermal analysis (DTA) of Zn adsorbed samples of AA and PAA.

To further explore the impact of metal ion binding on the thermal stability of the adsorbent, TGA analysis was conducted on Zn(ii)-adsorbed AA and PAA. The results showed that the thermal decomposition patterns of the Zn(ii)-adsorbed AA and PAA closely resembled those of the pure polymers, with a notable difference in the percentage weight loss. Specifically, the weight loss was 68.44% for Zn(ii)-adsorbed AA and 52% for Zn(ii)-adsorbed PAA (Fig. 12a). These findings are consistent with the quantity of Zn(ii) adsorbed by both AA and PAA. Further, the DTA analysis of the Zn(ii)-adsorbed polymers revealed a clear shift in the maximum decomposition temperatures, from 252 °C to 257 °C for AA and from 244 °C to 251 °C for PAA (Fig. 12b). This shift is likely due to the coordination or adsorption of Zn(ii) ions on the surface of the adsorbents, which caused the onset of pyrolysis to occur at higher temperatures. The TGA and DTA results demonstrate that PAA retains stability up to ∼200 °C, with further mass loss associated with decomposition of functional groups and the polysaccharide backbone. After Zn(ii) adsorption, the onset of degradation shifts slightly, suggesting that metal–ligand coordination enhances structural rigidity. This improved stability is significant for environmental applications, where sorbents may undergo repeated adsorption–desorption cycles, drying, or moderate thermal treatments for regeneration. The data indicate that PAA can withstand such conditions without rapid degradation, supporting its potential for long-term use in water treatment or metal recovery processes.

**Fig. 13:**
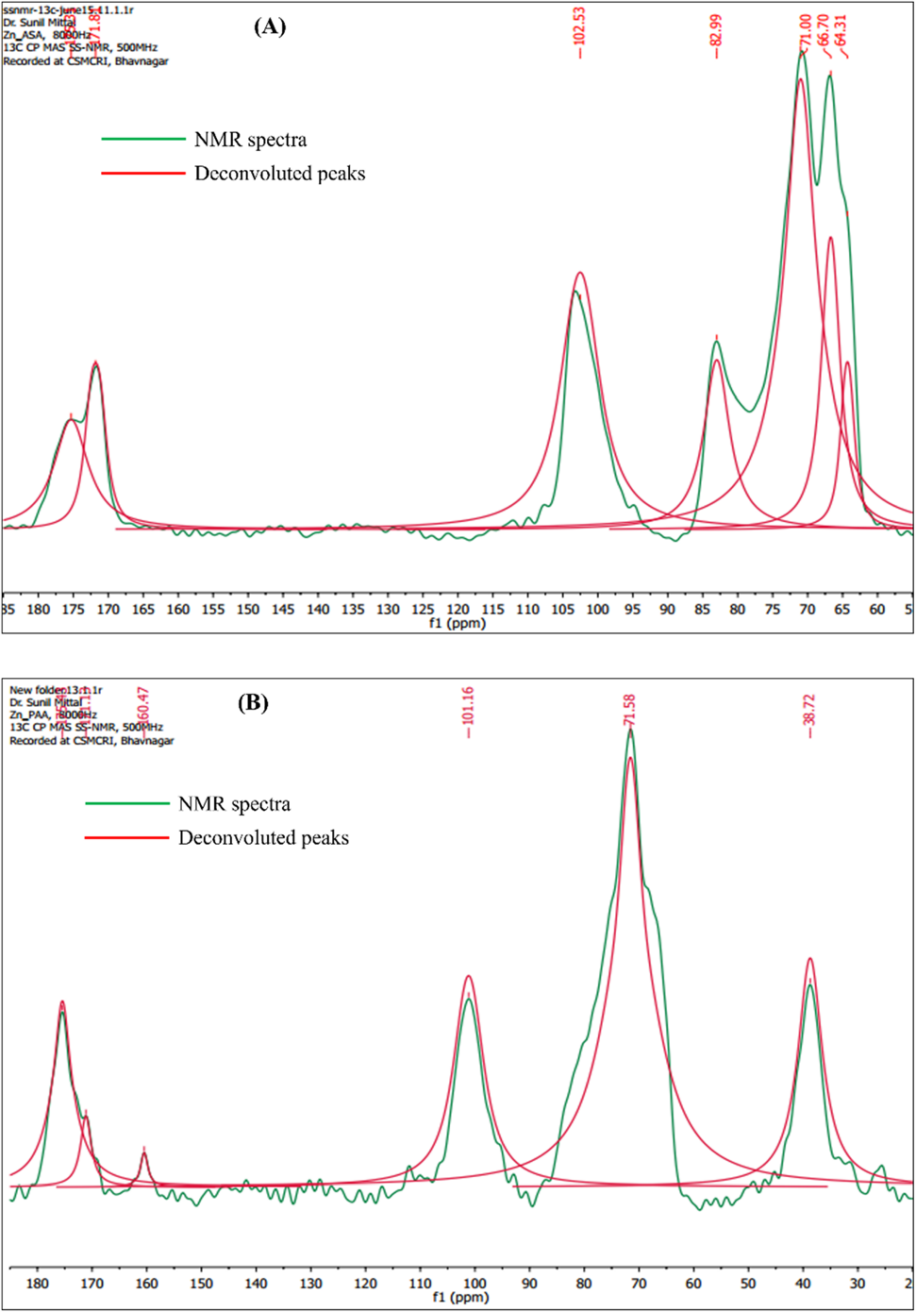
Global Spectral Deconvolution of CP-MAS ^13^C NMR spectrum of Zn adsorbed (a) AA; and (b) PAA.

The CP-MAS ^13^C NMR spectroscopic studies of Zn(ii) adsorbed AA, the carboxylate moiety of AA showed a remarkable shift with the appearance of two distinct peaks at *δ* 171.81 and *δ* 175.31 ppm which showed a strong but differentiated interaction between Zn(ii) and the two sub-units *i.e.* G and M (Fig. 13a). The intensities further indicated that the Zn(ii) interacted with M leading to a downfield shift of Δ*δ* 2.49 ppm in presence of Zn(ii), whereas, the M sub-unit showed a slight upfield shift of Δ*δ* 1.01 ppm, which indicated a feeble interaction of Zn(ii) with gluronic acid moieties in comparison to the mannuronic acid moieties in the substrate. On the other hand, CP-MAS ^13^C NMR spectra of Zn(ii) adsorbed PAA revealed downfield shift of the imine peak from *δ* 159.83 to 160.47 ppm. Apart from this amide carbonyl carbon also showed an upfield shift up to *δ* 171.13 ppm which is characteristic of Zn(ii) adsorption (Fig. 13b).

**Scheme 2:**
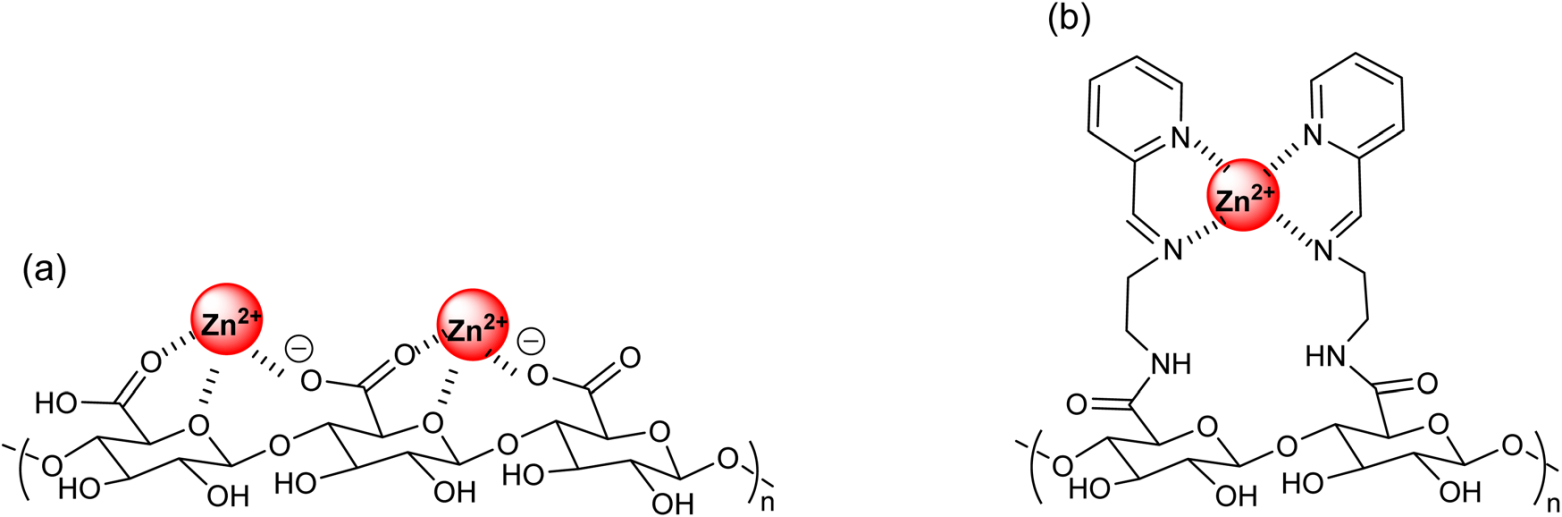
Proposed complexation of (a) Zn(ii) adsorbed AA; (b) Zn(ii) adsorbed PAA.

Based on the results of FTIR and CP-MAS ^13^CNMR, possible interaction of Zn(ii) with carboxyl group for AA along with that of amide/imine group in the case of PAA are represented as Scheme 2 and expected coordination for Zn(ii) ions with AA and PAA is supported with the spectral evidences obtained in this study. The enhanced uptake of Zn(ii) by PAA can be attributed to the new nitrogen functionalities introduced during modification. The imine groups (–C=N–) provide electron-rich nitrogen sites capable of coordinating Zn(ii) through lone-pair donation, while the amide groups (–CONH–) contribute both through carbonyl oxygen coordination and secondary interactions such as hydrogen bonding, which stabilize surface complexes. Together, these groups increase the density and diversity of active binding sites compared to native alginate, which primarily relies on carboxylate groups. The coexistence of imine and amide sites thus enables multiple coordination modes, explaining the higher sorption capacity and affinity observed for PAA.

### 3.4. Effect of interfering anions and competing cations on Zn(ii) removal by AA and PAA

**Fig. 14:**
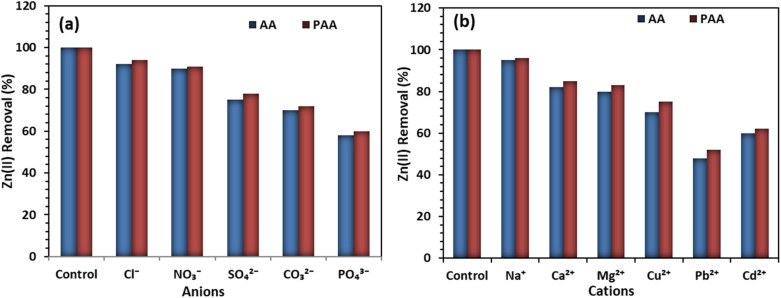
Effect of interfering anions (a); and competing cations (b), on the Zn(ii) removal by AA and PAA.

To simulate realistic aqueous conditions, the effect of common anions (Cl^−^, SO_4_^2−^, NO_3_^−^, CO_3_^2−^, and PO_4_^3−^) on Zn(ii) adsorption by AA and PAA was examined at the optimized pH (5.0), adsorbent dose (0.5 g L^−1^), and initial Zn(ii) concentration (100 mg L^−1^). The results indicated that the presence of background anions reduced Zn(ii) removal efficiency to varying extents. Weakly coordinating anions such as chloride and nitrate caused only a slight decrease (<10%) in adsorption, suggesting minimal competitive interactions. In contrast, strongly coordinating anions such as sulfate, carbonate, and phosphate significantly suppressed Zn(ii) uptake. In the presence of phosphate, the removal efficiency of Zn(ii) decreased by more than 40% for both AA and PAA, which can be attributed to the formation of stable Zn–phosphate complexes and competitive binding at surface functional groups. Similarly, carbonate and sulfate anions reduced Zn(ii) removal by 20–30%, primarily due to complexation in solution and electrostatic shielding effects (Fig. 14a).

The influence of common background cations (Na^+^, Ca^2+^, Mg^2+^, Cu^2+^, Pb^2+^, and Cd^2+^) on Zn(ii) removal by AA and PAA was also examined at the optimized conditions. The results revealed that the presence of monovalent cations such as Na^+^ had little impact on Zn(ii) uptake (<5% reduction), indicating negligible competition for adsorption sites. However, divalent alkaline earth metals (Ca^2+^, Mg^2+^) reduced Zn(ii) removal by 15–20%, mainly due to ionic strength effects and competition at carboxylate sites. Transition and heavy-metal cations exhibited stronger interference: Cu^2+^ and Cd^2+^ reduced Zn(ii) removal by 25–35%, while Pb^2+^ caused the greatest suppression, lowering Zn(ii) uptake by over 45% for both AA and PAA. This can be attributed to the higher affinity of Pb^2+^ for carboxylate and nitrogen donor groups, consistent with the Pb(ii) > Cu(ii) > Zn(ii) selectivity observed in the adsorption studies. These findings emphasize that the performance of AA and PAA is strongly influenced by competing divalent and multivalent cations, highlighting the need for selectivity assessments in real wastewater matrices (Fig. 14b).

As summarized in Table 4, PAA delivers Zn(ii) uptake comparable to many biopolymer-derived adsorbents (*e.g.*, CMC at approx. 10.8 mg g^−1^ and immobilized algal/alginates at approx. 22.2 mg g^−1^), and although certain engineered materials such as MnFe_2_O_4_/chitosan–Schiff base (289.9 mg g^−1^) or ZnO–C (189 mg g^−1^) show higher absolute capacities, they may pose environmental and cost challenges. Meanwhile, synthetic ion-exchange resins often require harsher regeneration and pose disposal concerns. By contrast, PAA offers a balanced performance with mild regeneration, biodegradability, and low toxicity, thus highlighting its potential as a sustainable and effective Zn(ii) sorbent.

**Table 4:** Comparison of Zn(ii) adsorption performance by various adsorbents

Adsorbent	Maximum Zn(ii) uptake (mg g^−1^)	Regeneration strategy	Sustainability/toxicity notes	Reference
MnFe_2_O_4_/chitosan-Schiff base nanocomposite	289.9	Magnetic separation/acid	Nanomaterial, possible leaching	50
Immobilized algal/calcium alginate beads	22.2	Acid desorption (*e.g.*, HCl, EDTA)	Biodegradable, biogenic	51
Ion-exchange resin	60	Strong acid/base	Synthetic, not biodegradable	52
ZnO–C nanocomposite	189	Acid regeneration	Engineered nanomaterial, possible leaching	53
Carboxymethyl cellulose (CMC)	10.8	Acid desorption (HCl)	Renewable, low toxicity	54
PAA	83.36	Mild acid or chelator (*e.g.*, EDTA)	Biodegradable, low toxicity	Present work

## 4. Conclusion

In conclusion, the stepwise coupling and condensation reaction successfully led to the formation of PAA by incorporating nitrogenous functional groups onto AA. Characterization techniques such as SEM-EDX, FTIR, ^13^C CP MAS SS-NMR, and TG-DTA demonstrated significant alterations in the surface and functional properties of the synthesized derivative compared to the parent bio-macromolecule. Batch adsorption experiments revealed that PAA exhibited a markedly higher adsorption efficiency for Zn(ii) compared to AA. A mechanistic analysis, based on FTIR and ^13^C CP-MAS NMR spectra of AA, PAA, and their Zn(ii) adsorbed forms, indicated the involvement of the carboxyl group in AA and the amide/imine group in PAA during Zn(ii) adsorption. This highlights that pyridine-2-imine derivatization of PAA enhances its adsorptive capability over AA due to the participation of the amide and imine groups in Zn(ii) binding, replacing the carboxyl group in AA. Both AA and PAA followed the Lagergren pseudo-second-order kinetic model for adsorption, with good agreement to both Langmuir and Freundlich adsorption isotherms, confirming the effectiveness and enhanced performance of PAA in Zn(ii) adsorption. The two-step functionalization of alginic acid employs mild conditions (ambient temperature, ∼24 h) and readily available reagents, making it suitable for scale-up. While DMF and MeOH were effective on the lab scale, greener solvents or recovery systems would improve industrial feasibility. The preservation of the polysaccharide backbone under these conditions ensures reproducibility, and process intensification (*e.g.*, continuous flow or microwave-assisted synthesis) could further enhance efficiency. Given the improved Zn(ii) uptake and the simplicity of the chemistry, this approach shows promising potential for large-scale applications in water treatment and metal recovery.

## Author contributions

Conceptualization: Upma Vaid, Sunil Mittal, J. Nagendra Babu, Harminder Singh, Sandeep Kumar; methodology: Upma Vaid, Sunil Mittal, J. Nagendra Babu, Harminder Singh; validation: Sunil Mittal, J. Nagendra Babu, Harminder Singh Sandeep Kumar; formal analysis: Upma Vaid, Sunil Mittal, J. Nagendra Babu, Rimzim Jasrotia, Harminder Singh, Sandeep Kumar; writing – original draft: Upma Vaid, Sunil Mittal, J. Nagendra Babu, Rimzim Jasrotia, Harminder Singh, Sandeep Kumar; supervision: Sunil Mittal, J. Nagendra Babu, Harminder Singh, Sandeep Kumar.

## Conflicts of interest

The authors declare no competing interests.

## Data Availability

The data generated and analyzed during this study are available from the corresponding author upon reasonable request. All relevant datasets, including characterization results and degradation performance metrics, can be provided to facilitate further research and verification of findings.
